# Teaching design thinking as a tool to address complex public health challenges in public health students: a case study

**DOI:** 10.1186/s12909-022-03334-6

**Published:** 2022-04-12

**Authors:** Carolyn Ingram, Tessa Langhans, Carla Perrotta

**Affiliations:** University College Dublin, Dublin, Ireland

**Keywords:** Design thinking, Human-centred design, Public health, Learning outcomes

## Abstract

**Background:**

Developing a public health workforce that can understand problems from a population perspective is essential in the design of impactful user-centred responses to current population health challenges. Design Thinking, a user-driven process for problem-defining and solution-finding, not only has utility in the field of public health but stands as a potential mechanism for developing critical skills -such as empathy, creativity and innovation- amongst future professionals. Though the literature reflects the use of DT across many health sciences disciplines, less research has been published on how students apply learned concepts using real-world challenges of their choice and what difficulties they face during the process.

**Methods:**

This case study evaluates achieved learning outcomes after the introduction of a design thinking block into post-graduate public health curriculum at the University College Dublin. Two independent assessors evaluated student learning outcomes and observed difficulties during the process by assessing group presentations to identify and understand any learning difficulties using an ad-hoc designed tool. The tool consisted of twelve items scored using a 5-point Likert scale. Student feedback, in the form of an online survey, was also analysed to determine their level of enjoyment, perceived learning outcomes and opinions on the course content.

**Results:**

The assessors evaluated thirteen DT group presentations and reports from 50 students. The groups chose a range of topics from socialization of college students during Covid-19 to mental health challenges in a low-income country. Independent assessment of assignments revealed that the highest scores were reached by groups who explored a challenge relevant to their own lives (more than 80% of total possible points versus 60% class average). The groups that explored challenges more distant to themselves struggled with problem finding with a mean score of 2.05 (SD ± 1.2) out of 5 in that domain. The greatest difficulties were observed in problem finding and ideation. Though most students found the design thinking block enjoyable and relevant to their education, they recommended that the DT block be a stand-alone module. Students recognized that groups who chose a familiar topic experienced fewer difficulties throughout the process.

**Conclusion:**

The study showed that DT learning outcomes were best achieved when students focused on challenges, they had either personally experienced or were familiar with. These findings provide insight for future iterations of DT workshops and support the teaching of user-centred approaches to future public health practitioners.

**Supplementary Information:**

The online version contains supplementary material available at 10.1186/s12909-022-03334-6.

## Background

Despite the recognition of the need for a Public Health workforce well equipped to lead innovative and tailored approaches to current challenges [1–3], public health courses focus their curricula on quantitative research methods, data interpretation, and theories of health promotion and policy, rarely providing students with the opportunity to engage in experiential learning.[3, 4] However, there is increased acknowledgement of the importance of including creativity and innovation courses in health sciences education.[5, 6] Design Thinking (DT) in particular, or human–centred design, is popular throughout health science disciplines as a method for students to gain creative skills.[7–9] The method is a user-driven process and is, in essence, a tool to find new problems or to re-define well known problems to design an impactful solution [10, 11]. It generally includes the following five steps: *“*empathize”, “define”, “ideate”, “prototype”, “test”. The “empathize” stage, during which the design team needs to explore the challenge from the perspective of the user, is particularly appealing in the design of public health interventions and policies, with the growing recognition that effective solutions must be driven and informed by the people whose lives they affect. In the last two decades, this methodology has been successfully applied to diverse areas including the redesign of clinical spaces -notably delivery rooms and children’s emergency departments-, the design of applications to improve disease management, and numerous applications in challenging, low-resource settings. [12–14]A recent pilot study described a two-hour non-mandatory DT seminar added to Public Health curricula that proved to be well-received by students who indicated that they understood the key elements of design thinking via a self-assessment tool. [15] However, the study did not explore learning outcomes beyond participants’ self-assessments. There has been significant research on the teaching of user-centred design tools that identifies difficulties experienced by students during their learning process. [10, 11, 16] Yet for students in health sciences disciplines there remains no published research -that we are aware of- on challenges observed during the assessment of learning outcomes as well as experiences learning DT.

During the academic years of 2019/2020 and 2020/2021, at the School of Public Health in the University College Dublin (UCD), we introduced a DT block into the Master of Public Health (MPH) curricula embedded in a module allocated to principles of healthcare finance and management. The rational for the inclusion of the DT block in this course stemmed from the need to rethink health systems and the call to accelerate the introduction of patient-centred health services. The module assessment included a team project in which students had to replicate four of the five DT steps (empathize, redefine the problem, ideate, prototype) by choosing a public health challenge, conducting an empathy stage, redefining the problem, brainstorming ideas, and pitching one idea that could have a positive impact on the problem.

To address the identified gap in the health education literature on the application of DT amongst students in health sciences disciplines, this manuscript presents a description of the observed difficulties students experienced during a final DT assessment as observed by two independent assessors, as well as students’ evaluations of the implementation of the course, their perceived achievement of learning outcomes, and their opinions and satisfaction with the design thinking block.

## Methods

The MPH at the University College Dublin is a one-year full-time 90‒credit program. In the academic years 2019/2020- 2020/2021, we introduced a three-week graded design-thinking block organized in a two-hour design thinking seminar facilitated by the UCD Innovation Academy and three weeks of teamwork leading to a graded assignment. The two-hour seminar involved working on solving a challenge using a topic unrelated to healthcare –reducing carbon footprint– through the Stanford design thinking framework (empathize, define, ideate, prototype, test) [17]. A detailed outline of the block including the learning outcomes is described in Table 1.

**Table 1:** Design Thinking Block Outline and Assessment (2019/2020; 2020/2021classes) Master of Public Health, Division of Public Health, School of Public Health, Physiotherapy and Sports Sciences, University College Dublin

Design Thinking Block: Stage	Components
DT seminar and Lectures (6 h in total)	**• Aims:** To describe the design thinking method and provide an overview of each of the recommended steps **•** Two-hour seminar dictated by a facilitator from the UCD innovation academy **•** Facilitator: non-healthcare background with experience facilitating design hackathons for diverse students’ cohorts **•** Students to complete the “Carbon footprint” challenge **•** Four hours of asynchronous lectures on leadership, design thinking in healthcare and empathy tools
Learning Outcomes	**•** To apply the DT method to a public health challenge or health service challenge of their choice **Specific learning outcomes by DT stage:** ✓ Empathy: ◦ The team identified and used a range of tools to conduct the empathy stage. Range of tools: a) literature review b) interviews c) observation d) surveys e) social media comments f) reports g) pictures/experience e) extreme users’ interviews ✓ Problem statement: ◦ The problem has been redefined or defined considering the empathy stage ◦ The problem statement addressed the main obstacle -as identified by the empathy stage- to achieve the desirable outcome- ✓ Ideation: ◦ How many divergent ideas proposed the team to address the problem? ✓ Overall assessment: ◦ The solution aligns with the problem, the problem statement, the empathy stage, and the ideation ✓ Communication: ◦ The team communicated the idea in a memorable way
Assignment	**• Step 1:** Identify one public health or healthcare management challenge of choice **• Step 2:** Self-group in teams of four to five **• Step 3:** Conduct an empathy stage – literature review, ethnography, semi-structured interviews, observations **• Step 4:** Define the problem in light of the empathy stage process **• Step 5:** Ideation: propose as many as divergent ideas as possible Students were given 3 weeks to complete the assignment with 1–2 feedback sessions as required
Pitch	**•** Students pitch their assignments and submit a short report on the process and lessons learnt
Module assessment based on Kirkpatrick Model	**• Level 1 – Reaction**: Overall satisfaction with the activity **• Level 2 – Learning Outcomes**: Achievement of learning outcomes during the course by the students and by two independent assessors **• Level 3 – Behaviour**: Intention to use the learning outcome in the workplace **• Level 4 – Action**

*DT* Design Theory

After the first face-to-face seminar (February 2020) the COVID-19 pandemic was declared and the first lock-down imposed in Ireland which impacted Higher Education [18]. Following the seminar, students had four hours of asynchronous online lectures on DT, teamwork, and the concept of psychological safety in teams, leadership styles, and innovation in health care. They also had one synchronic session on the rationale behind the use of DT in healthcare, the stages of the DT process, and the expected learning outcomes, totalling 6 h of teaching by the innovation academy tutors (Table 1). The UCD Innovation academy tutors who participated in the facilitation of the seminar have experience in facilitating DT seminars for interdisciplinary teams. Once they had completed the two-hour seminar, students worked in groups for three weeks on a design thinking assignment.

The assignment consisted of pitching an innovative idea to solve a public health challenge or health service challenge of choice by applying the design thinking approach. Due to time constraints, we did not ask students to prototype and test the idea. After the identification of a problem, each team needed to undertake the DT steps by completing an empathy stage (also call “immersion”) using diverse methods -literature review, observation, semi-structured interviews, interviews, surveys, social media surveys-, producing a problem statement through a problem finding process, brainstorming divergent ideas, identifying one idea, and pitching it to the class along with a short report on their process (Table 1).

Each team was offered two hours of feedback spread over the three weeks. The feedback was provided over Zoom by the module coordinator. Students were invited to discuss their difficulties during the process and the instructor provided feedback, ensuring that each team understood the steps required to complete the DT challenge.

Teams could choose the number of times they would meet to discuss their project and how they would meet. They also could choose the amount of time allocated over the three weeks to each DT stage.

During the second iteration (2020/2021), as there were still restrictions on face-to-face teaching on campus, the seminar and assignment switched to an online format. Trainers from the UCD Innovation Academy facilitated the introductory DT seminar as a two–hour online activity instead of a face–to–face seminar.

### Students’ characteristics and feedback

After the end of the second iteration, we invited both cohorts (2019/2020 and 2020/2021) to complete an online survey. An adjusted Kirkpatrick model was used to assess the module. (Table 1) [19, 20].

The online survey did not gather demographic data to avoid students’ identification. First, we asked them to indicate their experience in public health (fieldwork), research, working in teams, problem–based learning, creative thinking, and public speaking using a five-point Likert scale. We also included a question on their previous DT knowledge. The survey then addressed student satisfaction with the seminar and the assignment (level 1). To assess level 2 (learning outcomes from student perspectives), we asked students to rate their perceived knowledge on DT, empathy, brainstorming, and teamwork.

Regarding level 3 (future behaviour), we asked if students thought they would use the tool in the future (behaviour), if they would keep DT in the MPH curricula, and in which format.

To conclude, we asked students to leave any comments and recommendations for future iterations.

### Learning outcomes and observed difficulties by assessors

Assessment of the difficulties encountered by groups during each phase of the DT assignment (i.e., empathy, redefining the problem, ideation) was conducted by two assessors for the second iteration class (2020/2021 N = 50 students in 13 groups). To complete the assessment, and because of the lack of suitable rubrics or validated instruments, an ad-hoc scoring rubric was designed.

The assessment tool consisted of twelve items (supplement material). The first seven items matched the expected learning outcomes for the DT stages as provided to the students. Regarding empathy, assessors were instructed to consider whether the team identified and used a range of tools to understand the problem from the perspective of the users or those experiencing the challenge. The students were encouraged to “immerse” themselves in the problem by taking pictures, talking to users, conducting surveys, and/or analysing the existing literature. During the problem-finding and defining phase, assessors evaluated how teams redefined the problem considering the information gained in the empathy stage. During the brainstorming phase, assessors observed the number of divergent ideas proposed to address the problem. As for ideation, assessors observed whether groups struggled to propose a relevant idea that aligned with the problem statement and the empathy stage and had the potential to make a positive impact. Assessors also considered the memorability of the pitch. Finally, assessors were instructed to consider the level of difficulty observed during the stages of empathy, problem finding/defining, brainstorming, ideation, and communication.

### Statistical analysis

Evaluator’s assessments by DT domain were recoded from 5 to 1 (highest score = 5) for each team’s final assignment. Two overall evaluation scores were obtained for each team by summing individual item scores per assessor (12 scored items: 60 possible points). Cohen’s weighted kappa was used to measure agreement between overall evaluator scores and inter-evaluator reliability deemed sufficiently high to combine scores in subsequent analysis (κ_ w_ = 0.44, moderate agreement). The level of difficulty encountered during each phase of the DT process was summarized by calculating the mean, median, and standard deviation of all difficulties’ scores per theme.

## Results

The three, weekly recalls for the student satisfaction survey resulted in 66 responses, of which 56 (56/80 70% response rate with valid answers) responses met inclusion criteria and were included in the analysis. Of 56 respondents, 22 (22/30 73% response rate with valid answers) completed the DT Block in the 2019/2020 academic year (in-person seminar and group work), while 31 (31/50 62% response rate) completed the DT block in 2020/2021 (online). Three respondents did not indicate in which year they completed the DT block (5%).

Most students had little to no experience (Self-rated 1–2 out of 5) undertaking field work in public health (*N* = 33, 59.3%) or research (*N* = 28, 50.0%). While many indicated that they had moderate to substantial experience (Self-rated 4–5 out of 5) in teamwork (*N* = 42, 75.0%), problem-based learning (*N* = 31, 55.4%), creative thinking (*N* = 29, 51.7%) and public speaking (*N* = 24, 46.4%), there were no significant differences in experience levels between the two cohorts (Table 2 and 3). Only 10 students (17.9%) indicated that they were familiar with DT before undertaking the DT Block.

**Table 2:** MPH DT Survey – Cohorts 1 and 2—Respondent’s experience in DT relevant fields

Variable	Self-Rated Experience										
	**1**			**2**		**3**		**4**		**5**	
	**n**	**(%)**	**n**		**(%)**	**n**	**(%)**	**n**	**(%)**	**n**	**(%)**
Experience in											
Field work (*n* = 54)*	19	(35.2)	13		(24.1)	11	(20.4)	7	(13.0)	4	(7.4)
Research (*n* = 56)	11	(19.6)	17		(30.4)	14	(25.0)	9	(16.1)	5	(8.9)
Teamwork (*n* = 56)	2	(3.6)	3		(5.4)	9	(16.1)	22	(39.3)	20	(35.7)
Problem-based learning (*n* = 56)	5	(8.9)	8		(14.3)	12	(21.4)	21	(37.5)	10	(17.9)
Creative thinking (*n* = 56)	6	(10.7)	13		(23.2)	8	(14.3)	18	(32.1)	11	(19.6)
Public Speaking (*n* = 56)	3	(5.4)	8		(14.3)	19	(33.9)	18	(32.1)	8	(14.3)

*MPH* Master of Public Health, *DT* Design Theory

^*^denotes missing values (*N* = 2)

^a^Respondents were asked to rate experience on a scale of ‘1 – no experience’ to ‘5 – substantial experience

**Table 3:** Level of difficulties observed during the 2020/2021s iteration group assignments by DT domain

DT Phase	Mean Difficulties Score^a b^ (± SD)
Empathy	3.12 (1.04)
Problem Finding/Defining the problem	2.77 (1.31)
Brainstorming	3.31 (0.97)
Ideation	2.77 (0.90)
Communication	4.46 (0.52)

*DT* Design Theory

^a^Step 1: Group overall scores calculated out of 60 for each evaluator (*N* = 13 per evaluator). Cronbach’s weighted alphas calculated; Moderate level of Agreement identified (K_W_ = 0.44). Step 2: Mean evaluators’ difficulties scores calculated by DT phase and group (*N* = 13 per DT phase). Step 3. Overall Mean and SDs calculated by DT phase

^b^Scores from 1 (substantial observed difficulties) to 5 (no observed difficulties)

The second iteration of students (2020–2021) teamed-up in thirteen-groups of three to five participants according to their interests. The chosen topics included four challenges that were relevant to the students’ own lives (e.g. social life during Covid-19, technology overuse in college students during Covid-19) and broader topics (e.g. child obesity, child physical activity).

### Overall scores

Four teams obtained a high average score (> 69% of the total score), six teams a moderate average score (50%-60%), and two teams a lower score (< 50%). The teams with the highest scores chose topics that were familiar to them or that they had experienced.

### Observed difficulties by DT domain

The assessors identified the most difficulties during the finding/defining the problem and ideation phases of the DT assignment, with groups scoring only 2.77 points out of 5 on average (Table 3). Teams were slightly more at ease with the empathy (mean = 3.12) and brainstorming (mean = 3.31) components of the assignment. During the communication phase, assessors observed few to no difficulties at all (mean = 4.46). The four teams that chose a topic relevant to their own lives had less difficulty defining the problem, with an average score of 4.66 in comparison to other groups (2.05) (results not shown).

### Students’ satisfaction and perceived achievement of learning outcomes

#### Learning outcomes

In relation to learning outcomes, most students strongly agreed (*N* = 20, 35.7%) or somewhat agreed (*N* = 28, 50.0%) that they could now identify the steps of DT and understood the importance of empathy within Public Health practice. Most of the students believed that the DT block helped them to understand the key concepts outlined in the learning outcomes, particularly notions of brainstorming, problem finding and empathy (*N* = 50, 89.3%) (results not shown in table).

#### Student enjoyment of DT Block

After completing both the DT seminar and assignment, 46 students (46/54 = 85.2%) indicated that they enjoyed or mostly enjoy the block, while no students indicated that they did not enjoy it. Eight students (8/54 = 14.8%) indicated indifference. Most students (46/55 83.6%) specified that they would or probably would keep the DT block in the MPH Curricula. Students who participated in the face-to-face seminar (2019/2020 cohort) showed slightly higher enjoyment with both the seminar and assignment than those who undertook the online version (2020/2021) (Table 4). The majority felt that they would (*N* = 14, 25%) or probably would (*N* = 32, 57.1%) use DT in their futures as professionals.

**Table 4:** MPH DT Survey – Self-Rated Experience using a five-point Likert scale

In relation to the DT Block, the student	**2019/2020** (*n* = 22)		**2020/2021** (*n* = 30) *		**Significance**
	**Mean**	**(s.d.)**	**Mean**	**(s.d.)**	***p* value**^a^
Enjoyed the block ^b^	1.45	(0.67)	1.8	(0.76)	n.s
Recommends keeping it in the MPH Curricula^c^	1.55	(0.91)	1.67	(0.76)	0.043

*MPH* Master of Public Health, *DT* Design Theory, *s.d* Standard Deviation

^*^1 missing vaue

^a^Pearson’s Chi-Square test

^b^Students were asked to rate their enjoyment of the block on a scale of 1 (Definitely yes) – 5 (Definitely not)

^c^Students were asked if they recommended keeping the DT block in the curricula on a scale of ‘1 – Definitely yes’ to 5 – Definitely not’

#### Additional comments by students

Twenty-five students left additional comments validating the survey results. Interestingly, two students recognised how groups choosing familiar challenges performed better. One student mentioned that the assignment was challenging (“*It was the hardest assignment we did in the program*”) and many mentioned the limitations that the online format posed to completing the assignment (“*Don’t do this online again”*)*.*

## Discussion

Despite the difficulties faced due to distance learning, the introduction of DT into the MPH course was well-received by both cohorts, with many students viewing the content as relevant to their futures in Public Health. Students in the second iteration had a lower response rate and slightly lower satisfaction with the course. A possible explanation is the switch to online learning due to Covid-19; as suggested by the students this experiential learning is best achieved in a face-to-face format. Future iterations will clarify how the online experience decreased satisfaction and engagement with the module. We did not link the students' satisfaction responses to their group work performance to preserve the identity of the students. It is possible that students that presented fewer difficulties -as assessed by the evaluators- had higher satisfaction with the module.

Although literature on this topic is currently very limited, existing case studies that involve the integration of DT principles into educational curriculum and interdisciplinary events for healthcare professionals and students have proven beneficial [21, 22]. The research on DT suggest that it not only has the potential to provide user-driven insight in the design of healthcare products and services [11], but it also provides professionals with the skills to engage meaningfully with community members [9] and promotes creativity in both problem solving and defining.

The question remains as to how to effectively integrate instruction of DT into an already busy MPH curriculum – how many hours to dedicate to the topic, how to best achieve learning outcomes and the desirable level of learning. While a simple seminar on the DT process would be sufficient to introduce students to the key principles of the process and achieve superficial learning, the relevance of DT to Public Health calls for more in-depth and active learning to take place, leaving students with practical skills that they can employ in their futures as professionals. Most students who undertook this survey indicated that they were satisfied with their learning outcomes and that the unit helped them to understand the key concepts of cognitive bias, brainstorming, problem finding, empathy and psychological safety. Yet independent assessment of the assignments revealed that students had not completely grasped all of these concepts, with key difficulties identified during problem finding and ideation. The strengths of the DT approach stem from its emphasis on these processes, and thus they must be successfully taught if students are to truly grasp the methodology and utilize it effectively in the future.

### Empathy

Empathy, that is the ability to view the world from the perspective of another, is not only central to the DT process but to the entire field of Public Health. Yet, developing empathy in Public Health students is rarely discussed. Literature on teaching empathy to students in other fields, typically in the nursing and medical fields, suggests that experiential learning is the most effective way to develop empathy through education, with case-based learning, interviews, role-playing, community engagement and practical placement increasing students’ abilities to understand and consider the perspectives of others [23].This type of learning is relatively uncommon in post graduate public health programs, despite much of public health work being based in the field [22]. Although students in this course were introduced to different tools to conduct the empathy stage and encouraged to use them during the assignment, not all teams were able to apply these skills and practice empathizing with their users.

Other case studies that recount the teaching of DT to Public Health students and health professionals have drawn on active learning techniques during the empathy stage, with participants interviewing each other as part of the process, taking on the role of both the designer and the user [15, 24]. Interestingly, in the 2020/21 cohort, the three teams that encountered the fewest difficulties during the empathy phase of the assignment focused on exploring challenges faced by university students (Table 3), a population group that they themselves were a part of, giving them the ability to reflect on their own experiences, observe those around them, and interview or survey their peers – markers of a true empathy stage. This not only suggests that allowing students to experience the empathy stage from both sides, as the designer and user, increases their perceived value and understanding of the process, but also suggests that the increased involvement of community members themselves in the design process is beneficial to outcomes, an idea that is becoming increasingly relevant.

### Cognitive bias and problem defining

Runco (2014), defines a problem as “a situation with a goal and an obstacle”, or many interacting obstacles, and argues that a problem must be understood and defined in terms of those obstacles if a suitable solution is to be found [25]. Problem finding is inherent to science and discovery. As the famous Einstein quote states, “*Galileo formulated the problem of determining the velocity of light but did not solve it. The formulation of a problem is often more essential than its solution, which may be merely a matter of mathematical or experimental skill. To raise new questions, new possibilities, to regard old problems from a new angle, requires creative imagination and marks real advance in science*” [26]. The assessment of the 2020/21 DT Assignments (Table 3) revealed that teams struggled to translate the information uncovered in the empathy stage to the problem definition stage, with several groups maintaining their original idea of the problem, consequently constraining their understanding of the issue and limiting the quality of their proposed solutions. Effective problem solving depends on a student’s ability to approach the problem definition phase, dispelling their assumptions of the problem and finding new ways to explore the challenge at hand [6, 25]. Creativity, which is essential for this type of problem definition, is a process that involves significant cognitive flexibility, both divergent (free-flowing) and convergent (focused) thinking, and the use of associative and analogical thinking (the ability to understand new concepts in terms of something familiar). Dehaan (2009) suggests that these are cognitive functions which can be taught to university-level students [6] despite the fact that traditional methods of education, which are very structured and guided, tend to limit creativity [25]. Public Health teaching is very methodological and quantitative and teaches students to think in very particular ways, which perhaps explains why many students struggled to embrace the divergent thinking, cognitive flexibility and ambiguity needed in DT. As the MPH is a graduate program that attracts people from diverse educational backgrounds, it can be assumed that their methods of problem solving and creativity skills vary. Creative tendencies and methods of thought in students from different disciplines deserves further exploration. Qualitative studies that engage both students and professors suggest that less-stringent, open-ended assignments that are practical and based upon a domain that students are familiar with help develop creativity and enhance problem solving skills [2, 18, 19]. Additionally, group work and asking students to provide peer feedback are commonly cited as beneficial approaches to teaching students to take on other people’s perspectives and question their own assumptions and methods, linking in well with the empathy phase [27]. Exposing students to risk-taking and increasing their self-confidence and feelings of safety when voicing their ideas is also recognized as an important element when teaching creativity [6, 28]. Interestingly, the DT seminar and assignment themselves could be very useful when teaching students how to harness creativity, integrating many of the techniques just described.

Ensuring that optimal learning outcomes are achieved by numerous students will also depend on where and when DT is placed within the curriculum. Students from both cohorts at UCD indicated that DT would be better integrated into the Health Promotion module. Health promotion, which focuses on empowering, supporting and enabling people to take control over their own health and well-being, often addresses social and environmental factors outside of the traditional healthcare settings to prevent poor health outcomes [20]. DT could be used as a method to explore complex social challenges and present innovative initiatives for programs to address them. DT adds to the repertoire of tools already used by public health professionals in community-based participatory research (CBPR), allowing them to collaborate with local stakeholders to develop context-specific solutions to self-identified challenges [17], and could effectively be integrated into modules that teach these methods. The Thomas Jefferson University in Pennsylvania, USA, recently integrated a DT workshop into their Introduction to Public Health module [15], highlighting the utility of DT as a mechanism to introduce students to the concepts of empathy, communication, qualitative research, problem solving, and teamwork – skills that are essential for all public health practitioners and should be emphasized throughout the entire MPH curricula.

### Limitations

There are many limitations to this experience. First, the DT block was designed as a face-to-face seminar follow by three weeks of experiential group learning for the students. The constraints of COVID-19 restrictions on higher education meant that students conducted their assignment online which could have impacted their engagement, ability to conduct an empathy stage and to immerse themselves in their selected topics. Another significant limitation was the lack of an existing rubric for assessing learning outcomes in a DT exercise. Still, the developed ad-hoc rubric was able to capture differences across groups.

### Future recommendations

Future iterations of this block will need to facilitate empathy and problem finding learning processes. Solutions likely include either focusing on challenges experienced by students or incorporating user-partners as co-creators during the DT exercise. This research indicates that while students understood the general concepts of DT, groups encountered difficulties immersing themselves in and understanding the problem.

## Conclusion

The introduction of design thinking in the Curricula of the MPH was well received by students, who were satisfied with their learning outcomes and would keep the activity in the curricula. Analysis of completed assignments suggests the importance of immersing oneself in the problem to effectively identify a solution. This experience will inform new iterations of the DT activity.

## Supplementary Information

**Additional file 1. **

## Data Availability

The datasets used and/or analysed during the current study are available from the corresponding author on reasonable request.

## Abbreviations

DT: Design Thinking
MPH: Master of Public Health
UCD: University College Dublin
