# Supplementary material for: Teaching design thinking as a tool to address complex public health challenges in public health students: a case study
Source: BMC Med Educ. 2022 Apr 12;22:270. doi: 10.1186/s12909-022-03334-6 (PMC9002025; doi:10.1186/s12909-022-03334-6)
Supplement: Supplementary file 1 — Additional file 1. [file 12909_2022_3334_MOESM1_ESM.docx]

**2020-2021 Design Thinking group work Assessment tool**

| Domain | Description |
| --- | --- |
| Overall assessment | The pitched solution aligns with the problem, the problem statement, the empathy stage and the ideation |
| Empathy (DT learning outcome) | The team identified a range of tools to conduct the empathy stage.  Range of tools: a) literature review b) interviews c) observation d) surveys e) social media comments f) reports g) pictures/experience e) extreme users’ interviews |
| Problem statement | The problem has been redefined or defined considering the empathy stage |
| Problem statement | The problem statement addressed the main obstacle/user needs -as identified by the empathy stage- to achieve the desirable outcome |
| Ideation | How many divergent ideas (in number) proposed the team to address the problem? * |
| Ideation | Does the idea address the user need specified in the problem statement? |
| Communication:  Did the team communicated their idea in a memorably way? |  |
| Empathy | Observed difficulties by the assessors |
| Problem statement | Observed difficulties by the assessors |
| Brainstorming | Observed difficulties by the assessors |
| Ideation | Observed difficulties by the assessors |
| Communication | Observed difficulties by the assessors |
| Overall score | Sum of individual five points Likert scale |
